# 1012. Case Study. Complex Abdominal Wall Hernias After Skin Grafting for Necrotizing Injuries: Case Series and Reconstruction

**DOI:** 10.1093/jbcr/irag033.200

**Published:** 2026-04-06

**Authors:** Kyung Yoon, Vaisny Balamurali, Hilla Katz-Lichtenstein, Tara Ranjbar, Michael L Cooper

**Affiliations:** Northwell, Marietta, GA; Ross University School of Medicine, Staten Island, NY; Northwell, Staten Island, NY; Northwell, Staten Island, NY; Staten Island University Hospital Northwell Health, Staten Island, NY

## Abstract

**Patient Presentation (age range, injury details, relevant history):**

Four patients aged 53–72 developed full-thickness abdominal wall defects following necrotizing infections or surgical complications, including prior Hartmann’s procedure, necrotizing fasciitis after cosmetic surgery, abdominal compartment syndrome during endovascular AAA repair, and left flank necrotizing infection. All patients underwent serial surgical debridements and temporary split-thickness skin grafting, which provided coverage but failed to restore fascial and muscular integrity, ultimately resulting in large, symptomatic ventral hernias.

**Clinical Challenges:**

These cases presented multiple challenges. Extensive soft tissue and fascial loss created high-risk, contaminated wound beds, limiting reconstructive options. Patients required careful timing of definitive repair, balancing ongoing infection control, comorbidities, and wound healing. Restoration of functional abdominal wall integrity and prevention of recurrent hernias demanded meticulous multidisciplinary planning involving general surgery, plastic surgery, and wound care specialists.

**Management Approach:**

Initial management included serial debridements, temporary skin grafting, negative-pressure wound therapy, and selective use of bridging mesh to maintain abdominal domain. Definitive reconstruction was performed in a staged manner, utilizing synthetic or biologic mesh combined with autologous flap coverage, with component separation or tissue expansion applied as needed. Surgical planning was individualized based on defect size, degree of contamination, and patient-specific factors to optimize both structural and functional outcomes.

**Outcomes:**

All patients developed large hernias prior to definitive reconstruction. Following staged mesh and flap-based repair, abdominal wall integrity and dynamic function were restored. Complications included temporary mesh infection in one patient. Post-reconstruction, patients reported improved mobility and quality of life, with no recurrent hernias noted during follow-up.

**Lessons Learned:**

Skin grafting alone is insufficient for full-thickness abdominal wall defects. Staged reconstruction improves both structural and functional outcomes, and early multidisciplinary planning is critical. Reconstructive strategies should be tailored to the individual patient based on defect characteristics and comorbidities. Timely intervention reduces complications and recurrent hernias while enhancing long-term patient quality of life.

**Applicability to Practice:**

Skin grafts alone are insufficient for full-thickness defects.

Staged reconstruction improves structural and functional outcomes.

Early multidisciplinary planning is critical.

Reconstructive strategies should be individualized based on defect size, contamination, and patient factors.

Timely intervention reduces complications, recurrent hernias, and enhances patient quality of life.

